# Small-Scale Interventions Improving Literature Search Skills Among Resident Doctors: An Observational Study

**DOI:** 10.7759/cureus.85612

**Published:** 2025-06-09

**Authors:** Jan Drmota, Zoe Ng, Roland Fernandes, Ashish K Shrestha, Anang Pangeni

**Affiliations:** 1 General Surgery, East Kent Hospitals University NHS Foundation Trust, Ashford, GBR

**Keywords:** confidence, foundation programme, literature search skills, resident doctor, small scale intervention

## Abstract

Introduction

Literature search skills (LSS) are fundamental to research practices, writing scientific articles, quality improvement projects, and audits. There is no formal LSS teaching in postgraduate residency training in the United Kingdom (UK), and the literature lacks consensus on which LSS domains should be taught. The purpose of our pre-post observational study was to evaluate the improvement in confidence levels across selected LSS and views on the inclusion of LSS teaching in the postgraduate curriculum among resident doctors, following a small-scale intervention at our District General Hospital.

Methods

A two-hour workshop, delivered by the clinical library team and aimed at resident doctors in their first two years of UK residency, covered eight selected LSS domains, including defining a research question and screening abstracts. Pre- and post-workshop questionnaires assessed participants' confidence levels across the LSS domains using a five-point Likert scale. Confidence data (non-parametric) were analysed using a right-tailed Wilcoxon signed-rank test. Feedback on the course was collected separately to evaluate participant satisfaction and insights.

Results

For the included 22 participants, median pre-workshop confidence across the eight LSS domains ranged from 2.0 (IQR 1.0-3.3) to 3.0 (IQR 1.8-3.0). Post-workshop, median confidence ranged from 4.0 (IQR 4.0-5.0) to 4.5 (IQR 4.0-5.0). The right-tailed Wilcoxon signed-rank test revealed a statistically significant increase (p < 0.01) in confidence across all domains, with a large effect size (r > 0.8). All participants rated the workshop as highly useful and expressed interest in additional sessions, confirming its applicability to future practice.

Conclusion

Even a small-scale intervention improves LSS confidence across several domains, demonstrating the efficacy of such activities for resident doctors. This is key for broader professional training goals in the healthcare sector and should be integrated as part of formal training programmes.

## Introduction

Literature search skills (LSS) refer to the essential abilities required to effectively search for and evaluate published literature in order to answer research questions for academic work. LSS are essential for resident doctors in training who wish to contribute to research, engage in quality improvement projects, and perform clinical audits. These skills allow clinicians not only to stay current with the latest medical developments but also to provide evidence-based care and contribute to the evolving speciality they work in.

Within the United Kingdom’s (UK) Foundation Programme curriculum, first- and second-year resident doctors are required to complete a quality improvement project as part of the second Higher Level Outcome, titled “A valuable member of the healthcare workforce” [1]. Following on from this, resident doctors who wish to enter a speciality programme are further required to submit evidence that they have completed an audit, quality improvement project, or research publication to score competitively in the selection processes. This includes, among others, the Core Surgical Training Programme [2] and the Clinical Radiology Speciality Programme [3]. Once doctors enter speciality training, there is a continued expectation that they further conduct audit and research work [4], with the General Medical Council expecting resident doctors to “use evidence from (their) practice, including research, audit, […] and other quality improvement information, to reflect accurately on (their) performance” as part of their continued professional development [5].

The current UK resident doctor training curriculum does not include formal teaching sessions on LSS. Therefore, to develop competitive portfolios with academic accomplishments for career progression, resident doctors must independently develop widely accepted good research practices, which encompass defining research questions, data extraction and interpretation, and bias reduction [6]. There is a lack of published literature on the efficacy of implementing formal LSS teaching sessions in the UK resident doctor training curriculum, and on the views of foundation doctors regarding this potential implementation. The aim of this small-scale, pre-post intervention observational study is to evaluate the impact such an intervention can have on resident doctors’ confidence in LSS, and on their interest in having such a teaching session included in their formal training curriculum.

## Materials and methods

By conducting reflective tasks as first authors, and through informal conversations with fellow resident doctor colleagues, we recognised a gap in LSS among resident doctors at our District General Hospital (William Harvey Hospital, Ashford), which is part of East Kent Hospitals University NHS Foundation Trust - a teaching and training trust which comprises three hospitals in the UK National Health Service. With a regional surgical conference being organised at our hospital, we felt it was a timely opportunity to conduct our intervention, aiming to prompt and guide resident doctors to involve themselves.

To establish which aspects of literature searching are necessary for resident doctors to complete foundation training and further speciality training application academic requirements, a literature review of published articles and courses on literature searching in clinical practice was conducted [6-18], and our institution’s clinical library team was consulted. The output of this was formulated into eight LSS domains (Table 1). Subsequently, we developed a survey to assess our participants’ self-reported confidence with these LSS domains on a five-point Likert scale. The developed surveys (see Appendices) used were tested among authors and non-participating doctors for validity and to check for response relevance and clarity.

**Table 1:** List of LSS domains identified as most relevant for our intervention LSS, Literature search skills

LSS domains selected as relevant for intervention
Conducting a literature search
Defining a research question
Using databases
Formulating search terms
Screening and selecting abstracts
Appraising papers
Extracting data from papers
Managing references

The target population for our study was resident doctors in their first two years of residency who were part of the UK's Foundation Programme at our District General Hospital (n = 40). The relevance and necessity of learning these LSS were deemed highest for this selected resident doctor subgroup. A voluntary response sampling method was used, whereby an email invitation was sent to the selected resident doctors five days before the workshop, and those who attended the workshop and chose to complete the survey were included in the study. The pre-workshop survey was released to resident doctors who accepted the invitation on 28 November 2023, to be completed before attending the workshop. The workshop was held on 29 November 2023. The post-workshop survey was sent to participants at the end of the workshop, with a deadline to complete it by the end of 30 November 2023.

Subsequently, we collaborated with the clinical library team to develop and deliver a focused, two-hour workshop covering the selected LSS domains and tailored to our target resident doctor population. The workshop covered literature review structures, defining research questions, building search strategies, choosing databases, screening and selecting abstracts, analysing research paper results and discussions, guidance on how to access research papers, and correct referencing. The first hour and a half of the workshop was lecture-style teaching, with the last half hour focused on facilitated worked examples. There were four facilitators who observed and helped attendees perform these skills.

Using a post-intervention survey (Appendix 2), we reassessed participants' self-reported confidence in conducting literature searches on the same five-point Likert scale.

The data were collected through online Google Forms (Google, Inc., Mountain View, CA, USA), and the raw data were tabulated onto a Microsoft Excel sheet (Microsoft® Corp., Redmond, WA, USA). Using Python’s SciPy library, the Shapiro-Wilk test was used for parametric testing, and the Wilcoxon signed-rank test was used to assess significance and effect size.

## Results

In total, 26 individuals accepted the invitation, completed the pre-workshop survey (surveys completed between 28 November 2023 and 29 November 2023), and attended the workshop. This included medical students on placements and resident doctors of different grades (from foundation year 1 to speciality training doctors).

Of these, 22 participants satisfied the inclusion criteria of being junior resident doctors within their first two years of residency and part of the UK Foundation Training Programme. Four participants were excluded (two medical students and two higher trainees who were more than six years into their training beyond the Foundation Training Programme) from the data analysis, as they did not meet the inclusion criteria. The included participants had varying levels of prior exposure to literature search training, with the median number of prior sessions being 1 (IQR 0-2).

Post-workshop surveys were completed between 29 November 2023 and 30 November 2023 by all 26 attending participants, but only the 22 participants who satisfied the inclusion criteria were included in the study.

Survey results

The data of included participants (n = 22), for pre- and post-intervention confidence levels across the eight LSS domains, is summarised in Table 2. The data was analysed with the Shapiro-Wilk test, which confirmed it was non-parametric and not normally distributed. Thus, the right-tailed Wilcoxon signed-rank test was used to analyse the significance of the data. Improvements across all LSS domains were statistically significant.

**Table 2:** Median pre- and post-workshop confidence results (five-point Likert scale) for included 22 participants Statistical analysis was done using the right-tailed Wilcoxon signed-rank test. LSS, Literature search skills

LSS domain	Pre-workshop median confidence (IQR)	Post-workshop median confidence (IQR)	Wilcoxon signed-rank test (Z score)	Significance (p-value approximation)	Effect size (r)
Conducting a literature search	2.0 (1.8-3.0)	4.0 (4.0-5.0)	3.89	0.0001	0.87
Defining a research question	2.0 (1.0-3.3)	4.5 (4.0-5.0)	3.84	0.0001	0.86
Using databases	2.0 (1.8-3.0)	4.0 (4.0-5.0)	3.85	0.0001	0.84
Formulating search terms	2.0 (1.0-3.0)	4.0 (4.0-5.0)	4.08	<0.0001	0.87
Screening and selecting abstracts	3.0 (1.8-3.0)	4.0 (4.0-5.0)	3.60	0.0002	0.80
Appraising papers	2.0 (1.0-3.0)	4.0 (3.8-5.0)	3.76	0.0001	0.82
Extracting data from papers	2.0 (1.0-3.3)	4.0 (4.0-5.0)	3.65	0.0001	0.80
Managing references	2.0 (2.0-3.0)	4.0 (4.0-5.0)	3.79	<0.0001	0.87

Feedback on workshop utility

The usefulness of the session had a median rating of 5.0 out of 5 (IQR 4.0-5.0). Participants felt they would use the knowledge gained in their future practice, with a median rating of 5.0 out of 5 (IQR 4.0-5.0). All respondents (100%, n = 22) expressed interest in further sessions focusing on these skills, and all respondents (100%, n = 22) acknowledged the potential benefits of integrating these workshops into their formal training curriculum.

Sample free-text comments

Some of the sample free-text comments from participants are as follows: “Would have this every year. It would be amazing for F1s/F2s starting,” and “It would have been better if the session was longer.”

## Discussion

The data from the study highlight a statistically significant improvement in confidence across all aspects of LSS among resident doctors attending the workshop. These improvements underscore the effectiveness of practical, hands-on training delivered at the appropriate level. The marked improvement in confidence and overall satisfaction among participants supports the need for integrating such workshops into the core curriculum for resident doctors within the NHS Foundation Training Programme. This integration could substantially benefit their career development, improving their ability to undertake research, audits, and quality improvement projects effectively. The importance of these skills is further attested by the broad range of publications and courses available, which aim to improve literature searching skills in clinical practice [6-18]. Our study addresses the current gap in the literature and the lack of consensus regarding which LSS should be integrated into the UK Foundation Training curriculum to best support resident doctors' career progression.

A scoping review by Hirt et al. affirms the importance of good LSS and the limited application of these in practice [17]. There is no identified consensus on successful interventions that can achieve better LSS, and thus better research output. Our intervention contributes to this development, indicating that even small-scale interventions can lead to local improvements in confidence, which can be further developed. Ilic et al. conducted a randomised control study, where improved confidence after a single workshop did not translate to significant improvement in LSS [18]. However, small-scale interventions like ours can act as prompts to increase resident doctors’ exposure to LSS, and the improved confidence can serve as a foundation for their further improvement [19].

The confidence improvement and exposure our intervention provides is a key starting point for resident doctors with minimal to no previous exposure to LSS, to meet the portfolio requirements in their career progression [1-5].

Limitations

While our intervention shows a significant impact on our local trust, our data is limited to a small sample size of resident doctors who engaged during the timeframe of a surgical conference. Thus, attendees may have been more receptive to engaging with the intervention compared to the wider cohort of resident doctors. Furthermore, the small scale only highlights the importance of such interventions in increasing LSS confidence among resident doctors. The long-term effect of this intervention is beyond the scope of our study, as we cannot establish whether improved immediate confidence and knowledge translate into sustained real-world performance in literature searching, audit, quality improvement, or research work. Most participants had minimal exposure to previous LSS teaching sessions. However, the varying experience levels among attendees may act as a confounding factor in the study. To better evaluate participants’ improvements, an objective assessment tool should be used in addition to the self-reported confidence measures employed in our study. Further robust improvement work is necessary to fully evaluate this.

## Conclusions

The small-scale LSS workshop significantly enhanced the confidence of resident doctors in our hospital. The unanimous interest in further sessions and the recognition of the workshop’s relevance at the undergraduate level highlight the need for early and continued training in LSS. Integrating this training into the Foundation Programme curriculum can impact resident doctors’ readiness and capability to engage in learning and contribute to evidence-based medicine. This initiative, therefore, not only addresses an immediate educational need but also aligns with broader professional training goals in the healthcare sector.

## Appendices

Appendix 1: Pre-workshop survey

**Table 3:** Pre-workshop survey questions and possible responses

Question	Possible responses
Name?	Free-text response
Email?	Free-text response
Current speciality?	Free-text response
Current grade?	Free-text response
Current work location?	Free-text response
How many publications do you have?	Numeric response
How confident are you in conducting a literature search?	1 - Not confident; 2 - Low confidence; 3 - Neutral; 4 - Confident; 5 - Very confident
How confident are you in defining a research question?	1 - Not confident; 2 - Low confidence; 3 - Neutral; 4 - Confident; 5 - Very confident
How confident are you using databases?	1 - Not confident; 2 - Low confidence; 3 - Neutral; 4 - Confident; 5 - Very confident
How confident are you in formulating search terms for database searches?	1 - Not confident; 2 - Low confidence; 3 - Neutral; 4 - Confident; 5 - Very confident
How confident are you in screening & selecting abstracts?	1 - Not confident; 2 - Low confidence; 3 - Neutral; 4 - Confident; 5 - Very confident
How confident are you in appraising papers (for quality and biases)?	1 - Not confident; 2 - Low confidence; 3 - Neutral; 4 - Confident; 5 - Very confident
How confident are you in extracting data from research papers?	1 - Not confident; 2 - Low confidence; 3 - Neutral; 4 - Confident; 5 - Very confident
How confident are you in managing references?	1 - Not confident; 2 - Low confidence; 3 - Neutral; 4 - Confident; 5 - Very confident
How many literature search sessions did you have at university?	Numeric response
Do you think it would be beneficial to have literature search workshop sessions at your undergraduate level education?	Yes/No

Appendix 2: Post-workshop survey

**Table 4:** Post-workshop survey questions and possible responses

Question	Possible responses
Name?	Free-text response
Email?	Free-text response
How useful did you find this session?	1 - Not useful; 2 - Low usefulness; 3 - Neutral; 4 - Useful; 5 - Very useful
Was the session delivered at an appropriate level?	1 - Not appropriate level; 2 - Low appropriateness; 3 - Neutral; 4 - Appropriate; 5 - Very appropriate
How confident are you in conducting a literature search after attending this workshop?	1 - Not confident; 2 - Low confidence; 3 - Neutral; 4 - Confident; 5 - Very confident
How confident are you in defining a research question after attending this workshop?	1 - Not confident; 2 - Low confidence; 3 - Neutral; 4 - Confident; 5 - Very confident
How confident are you using databases after attending this workshop?	1 - Not confident; 2 - Low confidence; 3 - Neutral; 4 - Confident; 5 - Very confident
How confident are you in formulating search terms for database searches after attending this workshop?	1 - Not confident; 2 - Low confidence; 3 - Neutral; 4 - Confident; 5 - Very confident
How confident are you in screening & selecting abstracts after attending this workshop?	1 - Not confident; 2 - Low confidence; 3 - Neutral; 4 - Confident; 5 - Very confident
How confident are you in appraising papers (for quality and biases) after attending this workshop?	1 - Not confident; 2 - Low confidence; 3 - Neutral; 4 - Confident; 5 - Very confident
How confident are you in extracting data from research papers after attending this workshop?	1 - Not confident; 2 - Low confidence; 3 - Neutral; 4 - Confident; 5 - Very confident
How confident are you in managing references after attending this workshop?	1 - Not confident; 2 - Low confidence; 3 - Neutral; 4 - Confident; 5 - Very confident
Will you use the knowledge you gained in your future practice?	1 - Not likely; 2 - Low likelihood; 3 - Neutral; 4 - Likely; 5 - Very likely
What is the main take-home message you took from the workshop?	Free-text response
How did you find this session?	Free-text response
Would you want more sessions like this in the future?	Yes/No
How can we improve the session? Do you think these literature search workshops would be useful in your undergraduate-level education?	Free-text response
Any other comments you would like to add?	Free-text response
